# Analysis of the Effectiveness of Early Intervention on Carbapenem Antibiotic Use

**DOI:** 10.7759/cureus.86390

**Published:** 2025-06-19

**Authors:** Yoritake Sakoda, Takanori Matsumoto, Masaki Yamaguchi, Misako Tajiri, Kotaro Yoshida, Yasuki Maeno

**Affiliations:** 1 Infectious Diseases, St. Mary's Hospital, Kurume, JPN; 2 Pharmacy, St. Mary's Hospital, Kurume, JPN; 3 Safety and Infection Control, St. Mary's Hospital, Kurume, JPN; 4 Clinical Laboratory, St. Mary's Hospital, Kurume, JPN; 5 Medical Quality Management Center, St. Mary's Hospital, Kurume, JPN

**Keywords:** antimicrobial resistant, antimicrobial stewardship, antimicrobial use density, carbapenem, days of therapy, de-escalation

## Abstract

Introduction

The use of carbapenem antibiotics is a known risk factor for the emergence of carbapenem-resistant Enterobacteriaceae (CRE), a growing global public health concern. In this study, we focused on cases where carbapenems were selected as the initial empirical therapy and where early intervention strategies were implemented to assess and potentially modify such prescribing practices. Our primary aim was to evaluate changes in carbapenem usage following the initiation of early interventions and to determine whether these measures contributed to more appropriate antimicrobial use. Additionally, by analyzing cases subject to intervention, we sought to identify key factors that can help prevent the unnecessary initial selection of carbapenems.

Methods

We conducted a retrospective analysis of cases subjected to early intervention for carbapenem use over a one-year period from April 2024 to March 2025. In principle, early intervention involved clinical rounds or review of medical records within 24 hours of carbapenem initiation. When antibiotics were deemed necessary to be changed or discontinued, this was documented in the medical record, and feedback was provided. Interventions were classified into four categories: (1) no recommendation for change (appropriate use), (2) recommendation to switch to an alternative agent (change in empirical therapy), (3) recommendation for de-escalation, and (4) recommendation for discontinuation. For categories (2) to (4), we also collected data on whether the recommendations were accepted. Carbapenem use was assessed using days of therapy (DOT) and antimicrobial use density (AUD). Monthly trends in DOT and AUD before and after the start of the intervention program were analyzed. In addition, we evaluated annual changes in the use of carbapenems and other broad-spectrum antibiotics.

Results

Between April 2024 and March 2025, early interventions were conducted in 377 cases. Among these, 220 cases (58%) were deemed appropriate and required no change in therapy. The second most common category comprised 106 cases (28%) in which a switch to an alternative agent was recommended. In 33 cases (9%), de-escalation was suggested based on the identification of the causative pathogen, and antimicrobial susceptibility results were available at the time of intervention. In 18 cases (5%), no evidence of infection was found, and antibiotics were considered unnecessary. The acceptance rate of recommendations was generally favorable across all categories. Following the implementation of early intervention, both the DOT and AUD for carbapenems showed a notable decline.

Conclusion

Early intervention after prescribing carbapenem was associated with a reduction in both AUD and DOT, suggesting improved antimicrobial stewardship. These findings underscore the importance of appropriate empirical antibiotic selection in minimizing unnecessary carbapenem use. To curb the inappropriate initial use of carbapenems, it is essential to follow the fundamental principles of infectious disease management when selecting antibiotics and to accurately interpret culture and susceptibility data. Interventions and education focused on these areas are crucial for promoting responsible antimicrobial prescribing.

## Introduction

In recent years, the emergence of antimicrobial-resistant (AMR) organisms has become a global public health issue [1,2], and urgent countermeasures are needed. In May 2015, the World Health Organization (WHO) adopted the Global Action Plan on AMR, urging all member states to develop their own national action plans [3]. In response, Japan formulated its own National Action Plan on AMR in 2016 [4]. The most recent version, the National Action Plan on AMR 2023-2027, sets a performance target to reduce the defined daily doses per 1,000 patients per day of intravenous carbapenem use by 20% from the 2020 baseline by the year 2027 [5]. Carbapenems are broad-spectrum antibiotics that should be reserved for severe or resistant infections. However, their use is a recognized risk factor for the emergence of carbapenem-resistant Enterobacteriaceae (CRE), which poses a growing global threat [6]. According to data from the Japan Surveillance for Infection Prevention and Healthcare Epidemiology (J-SIPHE), our institution's carbapenem consumption is slightly higher than that of comparable hospitals. Moreover, our facility has previously experienced an outbreak caused by CRE. In light of the national AMR action plan and our institutional history, reducing carbapenem use has become an urgent priority.

One established approach to promoting the appropriate use of carbapenem antibiotics is intervention during prolonged therapy [7]. However, a considerable number of cases lack identifiable pathogens due to negative bacterial culture results, making de-escalation recommendations difficult. It has been reported that 20-40% of sepsis cases yield negative culture results [8-10], and in such cases, the duration of carbapenem therapy may be prolonged due to the inability to de-escalate. Furthermore, studies have shown that unnecessarily broad-spectrum empirical antibiotic therapy is associated with increased mortality [11]. These observations suggest that preventing the unnecessary initial selection of carbapenems may be of fundamental importance in antimicrobial stewardship.

We focused on intervening in cases where carbapenems were selected as the initial empirical antibiotic. In an effort to reduce overall carbapenem consumption, we initiated an early intervention program targeting the use of carbapenem antibiotics in April 2024. This study aimed to evaluate changes in carbapenem usage following the implementation of early intervention and to determine whether the intervention led to improved prescribing practices. In addition, by analyzing the cases in which interventions were performed, we sought to identify key factors that may help prevent the unnecessary initial selection of carbapenems.

## Materials and methods

At St. Mary’s Hospital (Kurume, Japan), we implemented an early intervention program targeting patients in whom carbapenem therapy was initiated, starting in April 2024. Our hospital is the core hospital of the Kurume medical region, which has a population of approximately 450,000. The hospital is a regional medical support hospital with 41 departments, 1,097 beds, and 2,400 staff, and plays a particularly important role in emergency medicine. Early intervention means that, as a rule, clinical rounds and medical record data are reviewed on cases within 24 hours of initiation of carbapenem antimicrobial treatment, and feedback is provided in the medical record if antimicrobials are deemed to need to be changed or discontinued. Notification of new carbapenem prescriptions was obtained via the hospital’s infection control surveillance system. Patients from the hematology department and pediatric patients were excluded from the intervention. Patients from the hematology department were excluded because they are often immunocompromised, and the use of carbapenems as initial therapy for infections is generally considered appropriate. In addition, pediatric patients were excluded because we did not implement any antimicrobial stewardship interventions in this population. Early interventions were conducted by infectious disease physicians and pharmacists specializing in antimicrobial stewardship. We retrospectively analyzed cases in which early intervention for carbapenem use was performed between April 2024 and March 2025. Each intervention was categorized into one of four groups: (1) no recommendation (appropriate use), (2) recommendation to switch to an alternative agent (modification of initial empirical therapy), (3) recommendation for de-escalation, and (4) recommendation for discontinuation. For categories (2) through (4), we collected data on whether the recommendation was accepted. Carbapenem usage was assessed by calculating days of therapy (DOT) and antimicrobial use density (AUD). Monthly changes in DOT and AUD before and after the implementation of the intervention were analyzed to assess trends. In addition to yearly evaluations of DOT and AUD for carbapenems, we also monitored changes in the use of other broad-spectrum antibiotics. This present study received approval from the Ethical Review Board of our hospital.

## Results

**Figure 1:**
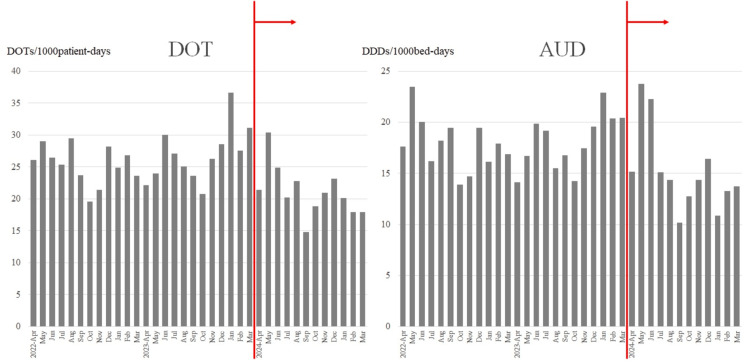
Monthly trends in the use of carbapenems The red line indicates the start of the intervention. DOT: days of therapy; AUD: antimicrobial use density; DDD: defined daily dose.

**Figure 2:**
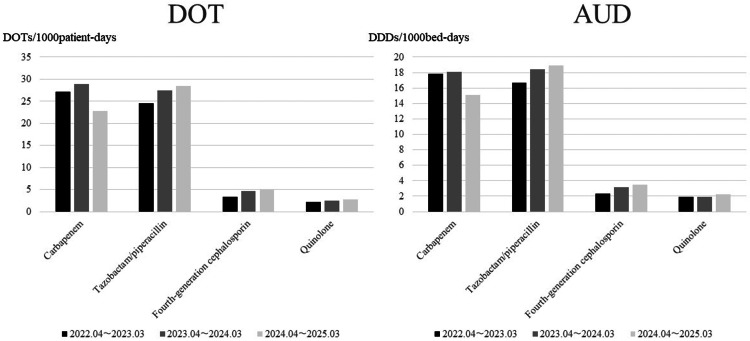
Yearly changes in the use of carbapenems and other broad-spectrum antibiotics DOT: days of therapy; AUD: antimicrobial use density; DDD: defined daily dose.

As shown in Table 1, early intervention was performed in 377 cases over the one-year period from April 2024 to March 2025. Among these, 220 cases (58%) were deemed appropriate, with no need for modification. The next most common category consisted of 106 cases (28%) in which a change to an alternative agent was recommended. In 33 cases (9%), de-escalation was proposed based on the identification of the causative pathogen and antimicrobial susceptibility test results at the time of intervention. In 18 cases (5%), no infection was confirmed, and antibiotics were considered unnecessary. The acceptance rate for the recommendations was relatively favorable across all categories. The changes in carbapenem usage following the initiation of early intervention are shown in Figure 1. After the intervention, both DOT and AUD gradually decreased and remained at reduced levels. Figure 2 illustrates the DOT and AUD for each year. In 2024, the DOT and AUD for carbapenems decreased by 21.2% and 17%, respectively. However, increases were observed in the use of piperacillin-tazobactam, fourth-generation cephalosporins, and quinolones.

**Table 1:** Results of interventions

Intervention	Number of cases, n (%) (N＝377)	Intervention acceptance	
		Accepted	Not Accepted
No recommendation	220 (58%)		
Recommendation to switch to an alternative agent	106 (28%)	88	18
Recommendation for de-escalation	33 (9%)	32	1
Recommendation for discontinuation	18 (5%)	15	3

## Discussion

In infectious disease management, it is considered essential to (1) understand the patient's background, (2) identify the infected organ, and (3) predict the causative bacteria. These steps represent the fundamental principles of infectious disease management. Adhering to these principles allows for the correct initial selection of antibiotics for cases where antimicrobial therapy is necessary. In the present study, the early interventions for carbapenem use were categorized into four main patterns (Table 1). In cases where a change in initial therapy was recommended, the causative pathogen had not been predicted in the majority of cases. This suggests that the principles of infectious disease management were not followed in these instances. In cases where the discontinuation of antibiotics was recommended, antibiotics were being administered for conditions that were not infections, such as malignancies or non-infectious inflammatory diseases, based on clinical examination or test results. This, too, was attributed to a failure to adhere to the principles of infectious disease management. To reduce carbapenem use, education is crucial. This education should not only focus on antimicrobial knowledge but also emphasize the principles of infectious disease management. In some cases, de-escalation was proposed because the causative pathogen had already been identified at the time of intervention. However, initial prescription in these cases was largely due to the improper interpretation of bacterial culture results and antimicrobial susceptibility tests. This highlights the need for education on the correct interpretation of test results. There were instances where, despite the availability of culture results, the attending physician did not acknowledge them. This suggests that additional methods, such as electronic health record reminders, may be necessary to ensure active confirmation of results, which is also important for patient safety.

In this study, the acceptance of recommendations was generally favorable. During the interventions, we recorded feedback in the patients' medical records. Additionally, we made an effort to clearly outline the thought process behind antibiotic selection. Feedback has been shown to have an educational effect on prescribing physicians [12]. After a recommendation is accepted, it is also important to monitor the patient’s progress together with the attending physician to ensure that the recommendation remains appropriate. We reviewed the cases where the recommendations were accepted, and no issues, such as worsening infections, were observed during the course of treatment.

Early intervention in the use of carbapenem antibiotics led to a reduction in both AUD and DOT. However, the prescription of tazobactam-piperacillin, quinolones, and fourth-generation cephalosporins increased. Such changes in overall hospital antibiotic prescriptions may be an inevitable consequence of reducing carbapenem use, but they could also lead to an increased risk of the emergence of new AMR organisms [13], warranting careful monitoring. To promote the proper use of antibiotics, appropriate interventions by the Antimicrobial Stewardship Team (AST) are essential. Among the recommended interventions by the AST are early monitoring and feedback on infectious disease treatment, as well as pre-approval of antibiotic use [14]. The early intervention in carbapenem use in this study corresponds to the former. Although pre-approval is not implemented at our institution, an antibiotic use notification system has been introduced as an alternative. The antibiotic use notification system is recommended in Japan, where there is a shortage of infectious disease specialists and infection control physicians, as a substitute for pre-approval [15]. This system involves targeting specific antibiotics for notification, auditing the reasons for their use, and proactively monitoring antibiotic treatment with feedback when necessary. Son et al. reported that early intervention in carbapenem use led to a reduction in its use, and further reductions were achieved with the introduction of pre-approval [16]. While pre-approval has clearly demonstrated reductions in antibiotic use and antimicrobial resistance [17,18], the evidence supporting the effectiveness of the antibiotic use notification system remains insufficient [19]. Moving forward, introducing pre-approval at our institution may further reduce the use of carbapenems and other antibiotics.

This study has several limitations. The baseline characteristics and clinical diagnoses of patients who received carbapenem treatment were not compared before and after the intervention. Additionally, while the usage of carbapenem antibiotics was assessed, an evaluation of outcomes was not conducted. Outcome evaluation in antimicrobial stewardship is challenging due to various confounding factors. Currently, we are considering the incidence of carbapenem-resistant *Pseudomonas aeruginosa* as an outcome measure, but long-term observation is necessary. The effects observed in this study cannot be guaranteed to be sustained, as the post-intervention period was relatively short. Long-term follow-up will be needed to assess the durability of these effects.

## Conclusions

Early intervention in the use of carbapenem antibiotics led to a reduction in both AUD and DOT. This study highlights the importance of appropriate initial antibiotic selection in efforts to reduce carbapenem consumption. To prevent the unnecessary empirical use of carbapenems, it is essential to adhere to the fundamental principles of infectious disease management when selecting antibiotics and to accurately interpret culture and antimicrobial susceptibility test results. Targeted interventions and education focused on these aspects are crucial for promoting appropriate antimicrobial use.
